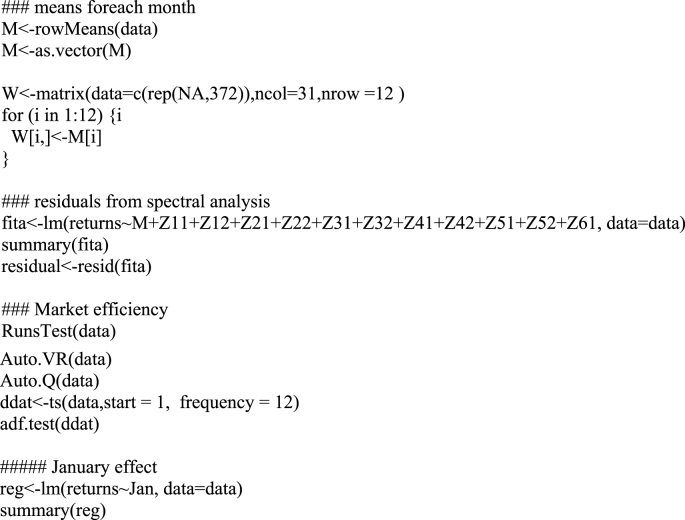# Comparative analysis of stochastic seasonality, January effect and market efficiency between emerging and industrialized markets

**DOI:** 10.1016/j.heliyon.2024.e28301

**Published:** 2024-03-24

**Authors:** Nancy Eduah, Godwin Debrah, Emmanuel Kojo Aidoo, Felix O. Mettle

**Affiliations:** a Department of Statistics and Actuarial Science, University of Ghana, Ghana; b Department of Computer Science and Information Systems, Ashesi University, Ghana

**Keywords:** Stochastic seasonality, January effect, Market efficiency, Emerging, Industrialized

## Abstract

This study investigates whether there are significant differences in investment returns between emerging markets and industrialized markets in terms of stochastic seasonality, January effect and market efficiency. Data on investments, and returns for nine emerging countries and eleven industrialized countries spanning January 1990 to December 2020, were obtained from the Organization for Economic Cooperation and Development (OECD). The spectral nonparametric test was used to determine the presence of stochastic seasonality for each market while the regression test was used to determine the presence of January effect. In the case of determining the efficiency status of the markets, the variance ratio test and the runs test were used. In cases where there appeared to be differences between the two types of market, Fisher's exact test was used to verify such differences. The results show no significant differences between the two types of markets in terms of seasonality, January effect and efficiency statuses. Apart from Brazil which recorded stochastic seasonality, all others are not stochastically seasonal. In the case of the January effect, it was a mixed bag; five emerging markets had January effect while two industrialized markets had January effect.

## 1 Introduction

Most existing time series models are based on the assumption of the Gaussian white noise series [1]. When working with time series, there are some important considerations that one must make. First, whether the series is stationary, second, is whether there is a seasonality component and third, is whether the series is autocorrelated or not. Seasonality is defined as a pattern of regular and repeated fluctuations in time series during a period of less than a year [2]. Testing for seasonality entails constructing a model for the seasonal and non-seasonal components of a time series and determining whether the seasonal component's parameters are significant over time. A seasonal component may either be deterministic or stochastic in nature. A deterministic seasonal component is easier to analyse as compared to a stochastic component [3]. A deterministic seasonal component is predictable while the stochastic is not.

An efficient market, according to Jensen [4] is one in which prices reflect information to the point where the marginal advantages of acting on information do not outweigh the marginal cost of obtaining information. A market is efficient when it follows a random walk. The concept of the random walk was introduced by Bachelier [5]. The efficiency status of markets is assessed by testing whether the returns follow the random walk hypothesis [6,7]. In general, the ideal market according to Fama [8] is one in which prices provide accurate signals for resource allocation. That is, one in which firms can make production-investment decisions and investors can choose among the securities that represent ownership of firms' activities, assuming that security prices depict all available information at any given time. There are three forms of an efficient market, namely, weak form efficiency, semi-strong form efficiency and strong form efficiency. With the weak form, current financial asset values, incorporate all existing past financial information. This means that prices will move in a haphazard manner. The semi-weak form, on the other hand, assimilates the weak form and in addition, prices change swiftly and without bias to consider any new public information provided on the market. The strong form assumes that prices include all available information on a market including weak form and semi-weak form and all private information regarding a financial asset. An anomaly in market efficiency is the seasonality effect [9,10].

The January effect is a well-known example of a seasonality effect. The term ‘January effect’ implies the stock return in January is higher than the rest of the year [11,12]. Eyuboglu and Eyuboglu [13] and Agnani and Aray [14] are a few of the studies that believe the January effect exists. Other authors, on the other hand, claim that the January effect doesn't exist. In Indian stock markets, Dash, Dutta and Sabharwal [15] discovered favourable effects in November, August, and December, as well as a negative influence in March. June effect was also discovered by Ferdous [16] in Bangladesh, where large positive returns were observed in the month of June. Many authors throughout the literature attempted to detect January effect worldwide using various models such as regression [13,17], ARIMA, ARCH and GARCH models [18,19], and stochastic dominance (SD) analysis [14,20]. This paper uses regression model to test the January effect because it has been shown to be effective and easy to interpret throughout the literature [12].

The implications of the seasonality test and market efficiency for investors and financial markets are relevant. Time series are often subject to seasonal movements and these seasonal patterns tend to evolve. These patterns can easily be removed if the seasonal pattern is deterministic but becomes difficult if the pattern is stochastic and excess returns are not obtainable using information contained in the past movement of prices. Several studies have focused on the predictability of return in non-emerging markets using the variance ratio test and run tests. This indicates that the market returns can be easily predicted hence inefficient [[21], [22], [23]]. In contrast, Rehman et al. [24] also used random walk properties from variance ratio tests and runs tests and found that some emerging market returns exhibit random walk. While most results are focused on the seasonality status of non-emerging markets [[25], [26], [27]], the same cannot be said of emerging markets. These conflicting results have engendered a comparative assessment of the seasonality status between emerging and industrialized markets using spectral nonparametric tests by Busetti and Harvey [28] in addition to variance ratio tests and run tests. The spectral nonparametric test by Busetti and Harvey [28] is an improvement of the test by Canova and Hansen [29] due to the following reasons. The spectral nonparametric test corrects for serial correlation under the null hypothesis that the series is deterministic seasonality. Even though there are recent models for seasonality tests such as random forest-based approach and generalized seasonality test,the spectral nonparametric test method has a straightforward interpretation and is easy to compute, hence if the focus is only on testing for stochastic seasonality, there is no overwhelming reason for preferring a parametric test.

The paper also compared the market efficiency status and January effect status between the two markets (emerging and industrial markets). A country is said to have an emerging market if it is improving its economy to become more advanced [30]. An industrial market is that of a country with matured economy and evenly distributed economic level [31].

The rest of the paper is organized as follows. Section 2 reviews the relevant literature on Market efficiency. Section 3 contains the materials and methods which include the theoretical framework and analysis strategy. Section 4 follows with results and discussions while Section 5 as concludes the study.

## 2 Literature review

This section presents a review of literature pertaining to seasonality and market efficiency. The section consists of both theoretical and empirical literature reviews.

### 2.1 Theoretical review

The theoretical review of this study deals with theories or structures in place, explaining why the issue of market efficiency and seasonality, is linked to stock indexes and investment. And the relevance of this study to that perspective.

First of all, the theoretical section introduces and briefly discusses the concept of the efficient market hypothesis (EMH) and its three forms. And further talks about seasonality and its models.

#### 2.1.1 The efficient market hypothesis

The efficient market hypothesis was first introduced by Fama [8]. An efficient market is characterized as a market in which a large number of rational, profit-maximizing participants are actively competing, each attempting to forecast future market values of particular securities, and where current key information is nearly freely available to all participants [32].

Fama [8] also defined an effective market as one in which an investor can choose among the securities that represent ownership of firms' activities under the assumption that security prices at any time fully reflect all available information. The efficient market hypothesis (EMH) states that share prices of securities reflect all available information [33]. Fama tested the hypothesis by introducing methods such as fair game models, expected return models, and random walk theory. The hypothesis was founded on the following conditions:

1. Trading securities have no transaction costs.
2. Every market participant has access to all available information at no cost.
3. Everyone agrees on the significance of current information for each security's present price and future price distributions.

Under the EMH the information is neutral, meaning we cannot use the previous price to estimate the future return, and the price should be random. Investors are unable to speculate by purchasing inexpensive stocks or selling overvalued equities. They should invest in the stock market at a reasonable price.

##### 2.1.1.1 Weak form efficiency

With the weak-form theory of efficiency, stock prices already represent all information that can be gleaned from market trade data such as price history, trading volume, and short interest. According to this version of the theory, trend analysis is useless. Past stock price data is freely available and can be obtained for a small fee. According to the weak-form hypothesis, if such data ever provided trustworthy signals about future performance, all investors would have figured out how to profit from them by now. Because a buy signal, for example, would result in an immediate price increase, the signals eventually lose their significance as they become more commonly recognized.

Comparatively, the weak-form theory has been investigated the most. Ullah and Asghar [34] investigated the weak form of market efficiency of the (FTSE) Financial Times Stock Exchange Group −100 index and rejected the weak form of an efficient market hypothesis. Birǎu [35] conducted a study comparing the weak form of EMH in the Romanian and Hungarian capital markets, using daily data from January 2007 to December 2011, and using the BET and BET-C indexes for the Romanian stock market and the BUX and BUMX indices for the Hungarian stock market. They concluded that none of the countries has ineffective capital markets. At the same time, the Hungarian market's anomalies were less than the Romanian market's, which could be due to a difference in maturity levels.

Fama [8] extended his work on the weak form hypothesis, focusing on more general regions rather than just testing previous returns. The new weak-form hypothesis is dubbed "test for return predictability," and it incorporates the growing field of forecasting returns using variables such as dividend yields and interest rates.

##### 2.1.1.2 Semi-strong form efficiency

Semi-strong form efficiency, according to Fama, is concerned with whether price efficiency adjusts to additional information that is obviously publicly known. The semi-strong form includes the historical price, as well as some other information, such as fundamental data on the firm's product line, management quality, balance sheet composition, patents held, earnings predictions, and accounting practices [36]. If the semi-strong form holds, publicly available information will be included in the price, meaning the price will fully reflect publicly available information.

##### 2.1.1.3 Strong form efficiency

According to this form of efficiency, stock prices can reflect all essential information about a company, including information solely available to corporate insiders. Financial specialists or management of publicly traded companies are examples of insiders. Insiders with monopolistic access to information can create higher returns than the average, and they can also use the information before it reaches the general public to gain advantages. Fama [8] renamed the semi-strong form hypothesis as a test for private knowledge, with the goal of determining if business insiders have access to confidential information. The results of the test show that business insiders can earn more from secret information than ordinary investors. Fama also attempted to assess the ability of mutual fund and pension fund managers to create extraordinary profits at the same time. The findings show that professional investors can't make more money following company insiders in most cases, and exceptions are few.

### 2.2 Seasonality effect

According to Lobão [37], one of the most common patterns of market efficiency anomalies is the seasonality effect, which comprises a variety of time-related impacts. People try to define a specific time period or set of times to test the peculiar phenomenon of stock returns, then determine if there are any rules we can follow or any speculative possibilities we can take advantage of. January effect, day of the week effect, the month of the year impact, monthly effect, holiday effect, Monday effect, Weekend effect, turn of the year effect, and so on are all examples of calendar effects. In this study, our focus will be geared towards January Effect.

#### 2.2.1 January effect

January effect can be defined as a phenomenon whereby the stock market returns in January are larger than the rest of the year, and this is usually due to a significantly low return in December. This phenomenon destabilises the stock market prices.

Rozeff and Kinney [38] were among the first to investigate seasonality in the stock market using empirical research. They discovered that January's stock returns are considerably higher than the other eleven months. As a result, they opined that it was due to tax-loss selling. Following this, empirical research has discovered a high level of January seasonality in both domestic and international stock and money market performances. Branch [39] also discovered that stocks with poor returns the prior year had significant gains in January. To test the tax-loss selling hypothesis, Branch chose stocks whose prices had reached their lowest point in the last week of December (TLS).

#### 2.2.2 Models for testing seasonality

The methods and techniques for time series analysis can be categorized as parametric and non-parametric methods. Parametric and Non-Parametric methods can be used to test the existence of seasonality. According to Gautam and Singh [40] despite their similarities, both methods have their differences.

According to Tsagris et al. [41], the parametric test is the hypothesis test which provides generalizations for making statements about the mean of the parent population whiles the nonparametric test is defined as the hypothesis test which is not based on underlying assumptions, i.e. it does not require population's distribution to be denoted by specific parameters.

Tavor [42], employed both parametric and non-parametric methods to test for seasonality in the stock returns in some selected industrial countries. Since both methods yielded materially similar results, they presented only the results of the non-parametric test. They found statistical evidence of January effect in thirteen out of the seventeen stock markets of the industrialized countries studied.

##### 2.2.2.1 Modelling seasonality by parametric approach

The parametric approaches, according to Gautam and Singh [40], assume that the basic stochastic stationary process has a specific structural configuration that can be represented with a few parameters. Some parametric models are discussed below.

i. Autoregressive Integrated Moving Average (ARIMA)

The Box-Jenkins method of identifying an autoregressive-integrated-moving average (ARIMA) model by differencing, to obtain a stationary series and usingthe sample autocorrelation function to select the order of the autoregressive and moving average parts, is the traditional way of time series modelling. Because ARIMA modelling appears to be complicated, many economists are doubtful of it. The ARIMA method is subjected to criticisms of the procedure of model selection. Hipel et al. [43] stated that the approaches they proposed were largely for identifying basic models in big samples. The correlogram, also called the autocorrelation plot is helpful in identifying the right ARIMA model with several hundred observations. Most ARIMA models are nonlinear models and require the use of a nonlinear model fitting procedure.

ii. Exponential Smoothing (ES)

This involves generalizing exponentially weighted moving averages (EWMA) to add a trend component in forecasting and allowing effects of seasonality in time series forecasting. According to Yu et al. [44] EWMA was developed without the assistance of a well-defined statistical model, exponential smoothing techniques are characterized as ad-hoc. Sukparungsee et al. [45] also suggested a statistical basis for exponential smoothing based on World decomposition for a random walk with noise.

iii. Regression models

Regression models show how one or more predictor variables affect a response variable. Linear regression, CART regression trees (CART), and support vector regression (SVR) models are the most used machine learning regression approaches. The CART is a decision tree-based recursive tree-like approach for splitting input variables [40]. Regression trees have also been widely applied to a variety of regression problems, including the environment, electrical load, and neurology. Support vector machines identify the hyperplane that optimizes the margin between two classes, which is how the SVR is created. SVR has been used to forecast problems in a variety of industries, including financial time series, software management, traffic management, electric load forecasting, and many more.

##### 2.2.2.2 Modelling by non-parametric approach

In the last decade, non-parametric approaches have grown in popularity. Its success is based on its ability to learn through trial and error, as well as improving accuracy over time. It is a distribution-free model [40].

The Kruskal-Wallis test is a non-parametric test that is used to see if two samples are from the same distribution. The one-way analysis of variance is a parametric counterpart of the Kruskal-Wallis test [40]. When the Kruskal-Wallis test rejects the null hypothesis, at least one sample stochastically dominates another sample. The test does not reveal where stochastic dominance occurs or how many pairs of groups are involved. All months (or quarters, as the case may be) have the same mean, according to the null hypothesis. The test statistic has a χ2 distribution under this hypothesis.

### 2.3 Empirical studies

Wachtel [46] took the first initiative into stock price seasonality study in the New York Stock Exchange (NYSE). His research focused on the examination of the Dow Jones Industrial Average from 1927 to 1942. His study recorded a frequent abnormal rise in the indexes of the thirty stocks from December to January in eleven of the fifteen years he studied. Forecasts based solely on seasonal changes, neglecting all cycle and trend analyses have a high chance of being accurate.

Rozeff and Kinney [38] pioneered studies to investigate seasonality in the stock market using empirical research. They discovered that January's stock returns are considerably higher than the other eleven months. As a result, they theorized that it was due to tax-loss selling. Following this, empirical research has discovered that stock and money market returns in both domestic and international markets are very seasonal in January. Both studies indicate seasonal effects in the US stock market.

Branch [39] also found that stocks that had negative returns the previous year had large gains in January. Branch chose stocks whose prices had reached their lowest point in the last week of December to test the tax-loss selling hypothesis (TLS). He stated that the results support the tax-loss selling hypothesis since the returns of the selected stock increased significantly over the next four weeks.

Canova and Hansen [29] developed another set of tests to investigate the structural stability of seasonal patterns over time. They used data on stock returns on value-weighted indices for seven industrialized countries (Japan, United States, Germany, France, United Kingdom, Italy and Canada). This data collection was examined to see if the January effect and other abnormalities were present. With the exception of the United Kingdom and Germany, they concluded that all stock returns showed some type of seasonality. January returns had the most significant seasonal dummies. Furthermore, deterministic dummies do not adequately capture the relevance of seasonal variability in US macroeconomics, according to the three data sets studied.

Busetti and Harvey [28] used a modified Canova and Hansen [29] test to check for nonstationary stochastic seasonality. The non-parametric adjustment for serial correlation is based on estimates of the spectrum at seasonal frequencies, and this simplified version of the test statistic is demonstrated to have the same asymptotic distribution as the original distribution. The inclusion of trends, even when modified to accommodate structural breaks or the inclusion of regressors with non-seasonal unit roots, does not impact the distribution of the seasonality test results when the null hypothesis is true. They suggested a parametric version of the test, and Monte Carlo experiments were used to compare its performance to that of the nonparametric test. A test that allows for breaks in the seasonal trend was developed using an illustration from the series on U.K. weddings. Their research revealed that the asymptotic distribution is unaffected by the breakpoint. The Wald test for the significance of fixed seasonal dummies is contrasted to a broad test against any form of permanent seasonality, deterministic or stochastic. It should be noted that comparable techniques can be performed to discover trading-day effects. The proposed test statistics all exhibit asymptotic distributions that belong to the Cramér–von Mises family under the null hypothesis.

In a study to know the extent to which outliers persist in supplementing the Halloween effect, Haggard et al. [47], used the bootstrap test of seasonal differences in return skewness to test for seasonality. Data from Morgan Stanley Capital International was administered to a sample size of 37 countries. The countries were then divided into two [2] sub-periods. The median regression and influence vectors were used to examine the periods for a likely change between outliers and the Halloween effect. Calendar effects are an important factor to note in seasonality studies. It explains the behaviour of stock markets or the effects of any market anomaly related to the calendar. It was deduced that both analyses gave a consistent result with the steady importance of outliers in creating the Halloween effect. Also, the study provided evidence that non-Halloween returns are significantly more negatively skewed than Halloween period returns.

Heymans and Santana [21], investigate the Johannesburg Stock Exchange (JSE) efficiency using the automatic variance ratio test, the Chow–Denning joint variance ratio and the joint sign test. They concluded that the practice of active management by portfolio managers in the South African market seems to defy logic if one considers the fact that the JSE as a whole is at the very least weak-form efficient.

Phan and Pham [48] tested the adaptive market hypothesis in the two main Vietnamese stock exchanges, namely Ho Chi Minh City Stock Exchange (HSX) and Hanoi Stock Exchange (HNX), by measuring the relationship between current stock returns and historical stock returns. In particular, the tests employed are the automatic variance ratio test (“AVR”), the automatic portmanteau test (“AP”), the generalized spectral test (“GS”), and the time-varying autoregressive (TV-AR) approach. The empirical results validate the adaptive market hypothesis in the Vietnamese stock market. Furthermore, the results suggest that the evolution of HSX has served as an important factor in the adaptive market hypothesis.

Ozkan [23], investigates the impact of the novel coronavirus (COVID-19) pandemic on stock market efficiency for six hard-hit developed countries, namely, the United States (US), Spain, the United Kingdom (UK), Italy, France, and Germany. Applying the wild bootstrap automatic variance ratio test on daily stock market data from July 29, 2019, to January 25, 2021, it was found that all stock markets used in this study deviated from market efficiency during some periods of the pandemic. Deviations from market efficiency are seen more in the stock markets of the US and UK during the COVID-19 outbreak than in other stock markets. The findings of this study indicate an increasing chance for stock price predictions and abnormal returns during the COVID-19 pandemic.

## 3 Materials and methods

This section discusses the analysis strategy used to address the objectives of the study and entails the models used in analysing the data such as spectral non-parametric test for stochastic seasonality, run test and variance ratio test.

### 3.1 Analysis strategy

This study made use of data from the database of the Organization for Economic Cooperation and Development (OECD). A sample of 20 countries (9 emerging and 11 industrialized markets) was used. The sample was based on the availability of data within the study time frame (1990–2020). The emerging markets used in the study are Brazil, India, Ireland, Israel, Korea, Mexico, Portugal, South Africa and Turkey; while the industrialized markets used are, Austria, Canada, Denmark, France, Germany, Netherlands, the UK and USA. Monthly data of stock returns for all 20 countries starting from January 1990 to December 2020 were obtained from the OECD.

The study used the spectral non-parametric seasonality test by Busetti and Harvey [28] at lags 4 and 8 (i.e., m=4 and m=8) as indicated early on to assess the presence or otherwise of stochastic seasonality. The independent variable used $X_{t}$ in equation [2] is a vector of length T=372 whose elements are the corresponding repetitions of the average return for each of the months across the study period for each market. The spectral non-parametric seasonality test was employed because according to Busetti and Harvey [28], parametric tests are attractive within the context of model-building exercise, but if the sole focus is on testing for stochastic seasonality, then there is no overwhelming case for preferring them to nonparametric tests. Also, the asymptotic null distribution of the test statistic is the same as that for the parametric test. Moreover, the presence of the January effect was tested for each country using the simple regression test, while that for market efficiency was tested for each country using the Variance Ratio test and the Runs test. The variance ratio test was used because it had been shown to have optimal power over the alternative hypothesis [7], whiles run tests were used for confirmation. In situations (i.e., seasonality status, January effect status and market efficiency status) where there appear to be differences between emerging markets and industrialized markets, Fisher's exact test was carried out to establish the significance or otherwise of the differences.

Busetti and Harvey [28] were of the view that taking the first difference is likely to be a good strategy for testing for the presence of stochastic seasonality in most economic time series. To assess this stand, the study after using the first differences for the seasonality test for all the twenty countries, also used the non-differenced data for ten countries (four emerging markets and six industrial markets) for the test. Before these tests, descriptive analysis was performed on the market returns of all the selected countries. In situations where the analysis involves laborious computations, R codes (see the appendix) were used while for the easy ones, simple excel formulae were used.

### 3.2 Theoretical framework

This section forms the basis of the methods used and involves the testing framework, the locally best invariant test, and the spectral nonparametric test which is the main statistical tool used in the study.

#### 3.2.1 The testing framework

Following the work of Canova and Hansen [29], let $y_{t}$ be a scalar time series and let s represent the number of seasons in a year and let $Z_{t}={\left(z_{it}^{\prime },\ldots ,{z^{\prime }}_{\left[s/2\right]t}\right)}^{\prime }$ be an (s−1) vector of trigonometric seasonal variables, that is $z_{it}=\left(\cos\;2\pi jt/s,\sin\;2\pi jt/s\right)\text{,}$ $j=1,2,\ldots ,s^{*}$, where $s^{*}=s/2-1$ if s is even or s/2 if s is odd,

Whereas $z_{\left[s/2\right]t}={\left(-1\right)}^{t}$ if s is even. The $j^{th}$ pair of the trigonometric terms, $z_{it}$, corresponds to the $j^{th}$ harmonic seasonal frequency, $\lambda_{j}\equiv 2\pi jt/s\text{,}$ $j=1,2,\ldots ,s^{*}$. When s is even, the last element of $Z_{t}$, $z_{\left[s/2\right]t}$ corresponds to the Nyquist frequency $\lambda_{\left[s/2\right]}\equiv \pi$.

The test against stochastic seasonality is developed in the context of the following unobserved component model from equation [[1], [2], [3], [4]].

(1) $$y_{t}=\mu_{t}+s_{t}+\varepsilon_{t},t=1,2,\ldots ,T$$

(2) $$\mu_{t}=X_{t}^{\prime }\beta \text{,}$$

(3) $$s_{t}=Z_{t}^{\prime }\gamma_{t}\text{,}$$

and

(4) $$A^{\prime }\gamma_{t}=A^{\prime }\gamma_{t-1}+\kappa_{t\;}\text{,}$$

where $X_{t}$ is a p×1 vector of linearly independent deterministic regressors with associated coefficient vector β**,** $s_{t}$ is the time varying seasonal component with $Z_{t}$ as defined earlier, A is a known (s−1)×k selection matrix with k≤s−1 and $\varepsilon_{t}$ and $\kappa_{t\;}$ are mutually uncorrelated mean 0 distribution with variance $\sigma_{\varepsilon }^{2}$ and $\sigma_{\kappa }^{2}W$. The initial $\gamma_{0}$ is assumed to be fixed.

The rationale is to test the null hypothesis that the seasonal component is deterministic, $H_{0}:\sigma_{\kappa }^{2}=0$, against the alternative that it has unit root behaviour, $H_{1}:\sigma_{\kappa }^{2}>0$. Following Canova and Hansen [29] as cited by Busetti and Harvey [28], the matrix A is used to formulate tests at subsets of the seasonal frequencies $\lambda_{1},\lambda_{2},\ldots ,\lambda_{\left[s/2\right]}$. If the test is to be carried out at the single frequency $\lambda_{j}$, then we let $A_{j}$ denote the ${\left(2j-1\right)}^{\text{th}}$ and ${\left(2j\right)}^{\text{th}}$ columns of $I_{s-1}$ for j<s/2 and the ${\left(2j-1\right)}^{\text{th}}$ column of $I_{s-1}$ if j=s/2; $I_{k}$ denote an identity matrix of dimension k when all of the seasonal frequencies are considered, $A=I_{s-1}$.

#### 3.2.2 Locally best invariant test

In the views of Busetti and Harvey [28], the locally best invariant (LBI) test for the null hypothesis of deterministic seasonality under Gaussianity for the equation [[1], [2], [3], [4]] can easily be obtained from the results of King and Hillier [49].

Now, in the manner of Busetti and Harvey [28], let $e_{t},t=1,2,\ldots ,T$ be the ordinary least squares (OLS) residuals from regressing $y_{t}$ on $\left(X_{t}^{\prime }{,Z}_{t}^{\prime }\right)$ and let ${\hat{\sigma }}^{2}=T^{-2}\sum_{t=1}^{T}e_{t}^{2}$ be the sample variance. Also, let $a_{j}=1$ if i=s/2 and $a_{j}=2$ otherwise. Then considering each seasonal frequency $\lambda_{j}$ (j=1,2,…,s/2) in turn, i. e $A=A_{i}$ in equation [[1], [2], [3], [4]], the LBI test statistic for testing $H_{0}:\sigma_{\kappa }^{2}=0$ against $H_{1}:\sigma_{\kappa }^{2}>0$ is given in equation [5] as

(5) $$\omega_{j}=\frac{a_{j}}{T^{2}{\hat{\sigma }}^{2}}\sum_{t=1}^{T}\left[{\left(\sum_{i=1}^{t}e_{i}\;\cos\left(\lambda_{j}i\right)\right)}^{2}+{\left(\sum_{i=1}^{t}e_{i}\;\sin\left(\lambda_{j}i\right)\right)}^{2}\right]\text{,}$$

j=1,2,…,s/2

Under Gaussianity, large value of $\omega_{j}$ are inconsistent with the null hypothesis. It is worthy of note that when s is even, the statistic at the Nyquist frequency, $\omega_{s/2}$, can be written without the terms $e_{i}\;\sin\;\lambda_{s/2}i$, since they are identically zero.

As acknowledged by Busetti and Harvey [28], a complete test against stochastic seasonality at all frequencies (i.e., $A=I_{s-1}$), rejects for large values of the statistic which is the sum of the test statistics for each individual frequency, given by

(6) $$\omega =\sum_{j=1}^{s/2}\omega_{j}$$

This test, as observed by Busetti and Harvey (2003), is LBI for a model where under the alternative hypothesis, the coefficient corresponding to each seasonal frequency $\lambda_{j}$ ($j=1,2,\ldots ,s^{*}$) evolve as mutually independent random walks with variances $\sigma_{\kappa }^{2}$ and if s is even then the coefficient for frequency π is a random walk with variance $\sigma_{\kappa }^{2}/2$. Accordingly, W is an identity matrix unless s is even, in which case the last element in the main diagonal is 1/2.

When the null hypothesis is true, the asymptotic distributions of $\omega_{j}$ ($j=1,2,\ldots ,s^{*}$) and ω are Cramér–von Mises with $a_{j}$ ($j=1,2,\ldots ,s^{*}$) and s−1 degrees of freedom respectively. The ${\varepsilon_{t}}^{\prime }s$ need not be Gaussian; all that is required according to Busetti and Harvey [28] is that of being martingale differences satisfying the conditions of stationarity.

#### 3.2.3 Non-parametric correction for serial correlation based on the spectrum of seasonal frequencies

Busetti and Harvey [28] treated the serial correlation in the stationary component of equation [[1], [2], [3], [4]] nonparametrically by replacing the sample variance, ${\hat{\sigma }}^{2}$, in $\omega_{j}$ with an estimator of the spectrum of $\varepsilon_{t}$ at frequency $\lambda_{j}$. The result is a spectral nonparametric test statistic given in equation [7,8] as

(7) $$\omega_{j}\left(m\right)=\frac{a_{j}}{T^{2}\hat{g}\left(\lambda_{j};m\right)}\sum_{t=1}^{T}\left[{\left(\sum_{i=1}^{t}e_{i}\;\cos\left(\lambda_{j}i\right)\right)}^{2}+{\left(\sum_{i=1}^{t}e_{i}\;\sin\left(\lambda_{j}i\right)\right)}^{2}\right],j=1,2,\ldots ,\frac{s}{2}$$

where,

(8) $$\hat{g}\left(\lambda_{j};m\right)=\sum_{\tau =-m}^{m}w\left(\tau ,m\right){\hat{\gamma }}_{e}\left(\tau \right)\cos\;\lambda_{j}\tau$$

is the estimator of the spectral generating function, w(τ,m) is a weighting function or kernel, such as w(τ,m)=1−|τ|/(m+1), and ${\hat{\gamma }}_{e}\left(\tau \right)=T^{-1}\sum_{t=\tau +1}^{T}e_{t}e_{t-\tau }$ is the sample autocovariance of the OLS residuals at lag τ. See alternative options for the kernel w(.,.) by Andrews [50]. The spectral nonparametric statistic for testing all the seasonal frequencies is presented in equation [9] as

(9) $$\omega \left(m\right)=\sum_{j=1}^{s/2}\omega_{j}\left(m\right)$$

The asymptotic null distributions of the test statistics in equations [7,9] are respectively the same as those in equations [5,6,28]. The assumptions and prepositions together with the proofs upon which the spectral nonparametric test is based are discussed by Busetti and Harvey [28].

#### 3.2.4 Random walk and market efficiency

Let equations [8,9] represent a random walk with drift process

(10) $$X_{t}=X_{t-1}+\alpha +\epsilon_{t}$$

or

(11) $$S_{t}=\Delta X_{t}=X_{t-1}+\alpha +\epsilon_{t}$$

where,

$X_{t}$ represents the price index observed at time t

α represents an arbitrary drift parameter

$S_{t}$ represents the change in the index

$\epsilon_{t}$ represents the random disturbance term satisfying the condition $E\left(\epsilon_{t}\right)=0$, $\sigma_{\epsilon_{t}}^{2}$ is constant and $E\left(\epsilon_{t},\epsilon_{t-1}\right)=0\;\forall t\text{.}$

#### 3.2.5 The runs test

Randomness is very important in statistical inference specially in time series analysis. The run test is use to assess the assumption of the data being random. A run is defined as a series of consecutive negative or positive values [12]. The test statistic of the runs test is given in equation [12] as

(12) $$Z=\frac{T-\bar{T}}{S_{T}}$$

Where T is the observed number of runs, $\bar{T}$ is the expected number of runs and $S_{T}$ is the standard deviation of the number of runs.

The computational formulas for $\bar{T}$ and $S_{T}$ are given in equation [13,14] as

(13) $$\bar{T}=\frac{2n_{1}n_{2}}{n_{1}+n_{2}}+1$$

(14) $$S_{T}^{2}=\frac{2n_{1}n_{2}\left(2n_{1}n_{2}-n_{1}-n_{2}\right)}{{\left(n_{1}+n_{2}\right)}^{2}\left(n_{1}+n_{2}-1\right)}$$

where $n_{1}$ and $n_{2}$ denote the number of and negative values in the series.

Decision Rule**:**

Reject the null hypothesis if $\left|Z\right|>Z_{1-\frac{\alpha }{2}}$

Hypotheses:

$H_{0}:\text{Randomness}\;exists\;\left(independent\right)$

$H_{1}:Non\;\text{randomness}\;\left(dependent\right)$

Rejecting the null hypothesis is an implication that the data under consideration is not random at the given level of significance.

#### 3.2.6 Test for January effect

Given equation [15] as the return ($r_{t}$) model

(15) $$r_{t}=\mu +\beta \lambda +e_{i}$$

where μ is the constant and β is the coefficient of λ with λ=1 implying January effect and λ=0 implying no January effect, the appropriate hypotheses follows;

Hypotheses:

$H_{0}:\beta =0\;\left(no\;January\;effect\right)$

$H_{1}:\beta \neq 0\;\left(January\;effect\right)$

If there is January effect, it means that market is unstable.

#### 3.2.7 Unit root test

The study adopted Augmented Dickey Fuller (ADF) test for unit root.

Let $X_{t}$ be a time series generated in equation [16]

(16) $${\Delta X}_{t}=\alpha^{\prime \Delta_{t}}+\beta X_{t-1}+\epsilon_{t}$$

where $\epsilon_{t}$ is **I(0)** and may be heteroskedastic. The Phillips- Perron (PP) test correct for any serial correlation and heteroskedasticity in the error term $\epsilon_{t}$ which directly modifying the test statistic.

The modified statistics, denoted $Z_{t}$ in equation [17] and $Z_{\beta }$ in equation [18] are given by

(17) $$Z_{t}={\left(\frac{{\hat{\sigma }}^{2}}{{\hat{\lambda }}^{2}}\right)}^{\frac{1}{2}}\;\text{an}d\;T_{\beta }=-\frac{1}{2}\left(\frac{\hat{\lambda^{2}}-{\hat{\sigma }}^{2}}{{\hat{\lambda }}^{2}}\right)\times \left(\frac{T\cdot S.E\left(\hat{\beta }\right)}{{\hat{\sigma }}^{2}}\right)$$

But $Z_{\beta }=T_{\beta }-\frac{1}{2}\frac{T^{2}\cdot S.E\left(\hat{\beta }\right)\left({\hat{\lambda }}^{2}-{\hat{\sigma }}^{2}\right)}{\hat{\sigma }}\text{.}$

The terms ${\hat{\sigma }}^{2}$ in equation [18] and ${\hat{\lambda }}^{2}$ in equation [19] are constant estimates of the variance parameters.

(18) $$\sigma^{2}=\lim_{T\to \infty }\sum_{t=1}^{T}\frac{E\left({\varepsilon_{t}}^{2}\right)}{T}$$

(19) $$\lambda^{2}=\lim_{T\to \infty }\sum_{t=1}^{T}E\left(\frac{{S_{T}}^{2}}{T}\right)$$

where $S_{T}$ is expressed in equation [20] as

(20) $$S_{T}=\sum_{t=1}^{T}\varepsilon_{t}$$

The sample variance of the least square residual ${\hat{\varepsilon }}_{t}$ is a consistent estimator of ${\hat{\sigma }}^{2}$ and the Newey-West long run variance estimate of $\varepsilon_{t}$ using ${\hat{\varepsilon }}_{t}$ as a consistent estimate of ${\hat{\sigma }}^{2}$.

Hypotheses:

$H_{0}:\delta =0\;\left(there\;is\;a\;unit\;root,stochastic\right)$

$H_{1}:\delta <0\;\left(there\;is\;no\;unit\;root,stationary\right)$

#### 3.2.8 Variance ratio test

To test the null hypothesis that a given time series $X_{t}=y_{t}-y_{t-1}$ is the collection of iid observations, we define the variance ratio test of k period returns in equation [21] as

$$V\left(k\right)=\frac{\frac{\text{Var}\left(x_{t}+x_{t‐1}+\cdots +x_{t‐k+1}\right)}{k}}{\text{Var}\left(x_{t}\right)}$$

(21) $$=\frac{\text{Var}\left(y_{t}‐y_{t‐k}\right)/k}{\text{Var}\left(y_{t}‐y_{t‐1}\right)}=1+2\sum_{i=1}^{k}\left(\frac{\left(k‐1\right)}{k}\right)\rho_{i}$$

where, $\rho_{i}$ is the $i^{th}$ lag autocorrelation of $x_{t}$. V(k) is a particular linear combination of the first (k−1) autocorrelation coefficient with linearly declining weights [51].

The central idea of the variance ratio test is based on the observation that when returns are uncorrelated with respect to time, we expect that $Var\left(X_{t}+\ldots +X_{\left\{t-k+1\right\}}\right)=kV\left(X_{t}\right)$, that is V(k)=1. The null hypothesis of the ratio test as specified in equation [22] as

(22) $${\;H}_{0}:\rho_{1}=\cdots =\rho_{k}=0\text{,}$$

which implies that the returns are serially uncorrelated. A test can be constructed by considering statistic based on as estimator of V(k) in equation [23]. Thus,

(23) $$\gamma R\left(k\right)=\frac{{\hat{\sigma }}^{2}\left(k\right)}{{\hat{\sigma }}^{2}\left(1\right)}\text{,}$$

where ${\hat{\sigma }}^{2}\left(1\right)$ is an unbiased estimator of the one period return variance using the one period return $x_{t}$ and is defined in equation [24] as,

(24) $${\hat{\sigma }}^{2}\left(1\right)={\left(T-1\right)}^{-1}\sum_{t=1}^{T}{\left(x_{t}-\hat{\mu }\right)}^{2}$$

$$=\frac{1}{T-1}\sum_{t=1}^{T}{\left(y_{t}-y_{t-1}-\hat{\mu }\right)}^{2}$$

where $\hat{\mu }=\frac{\sum_{t=1}^{T}x_{t}}{T}$ is the estimated mean.

Hypotheses:

$H_{0}:{VR}_{\left(q\right)}=1\;\left(Unity\;under\;RWH\right)$

$H_{1}:{VR}_{\left(q\right)}\neq 1\;\left(No\;unity\;under\;RWH\right)$

## 4 Results and discussions

This section presents some descriptive statistics of the returns of all the markets under study over the period of investigation. Also presented are results of seasonality, January effect and market efficiency tests by type of market.

### 4.1 Descriptive statistics

Table 1 shows the nature of the stock market for the countries under observation. It presents the minimum, maximum, mean and standard deviation of the market returns of each country. The mean shows the central tendencies of the data while the standard deviation demonstrates the level of dispersion of the market returns.

**Table 1:** Descriptive statistics of investment returns by country.

Country	Min.	Max.	Mean	Std. Dev.
Austria	−0.3985	0.2015	0.0022	0.0567
Brazil	−0.8194	4.1800	0.0556	0.2616
Canada	−0.2500	0.1119	0.0040	0.0406
Switzerland	−0.1807	0.1421	0.0048	0.0399
Germany	−0.2816	0.1304	0.0033	0.0488
Denmark	−0.2686	0.1587	0.0067	0.0445
France	−0.2819	0.1100	0.0031	0.0468
UK	−0.2419	0.0999	0.0027	0.0371
India	−0.2752	0.3470	0.0111	0.0678
Ireland	−0.3179	0.1439	0.0039	0.0526
Israel	−0.2551	0.1335	0.0082	0.0508
Italy	−0.3069	0.1680	0.0017	0.0556
Japan	−0.2479	0.1336	−0.0013	0.0489
Korea	−0.2358	0.2072	0.0030	0.0627
Mexico	−0.2301	0.1849	0.0125	0.0599
Netherlands	−0.3046	0.1265	0.0040	0.0462
Portugal	−0.2209	0.1345	0.0036	0.0478
Turkey	−0.3525	0.5436	0.0241	0.1078
US	−0.2547	0.1193	0.0053	0.0375
South Africa	−0.2218	0.1235	0.0077	0.0442

From Tables 1 and it can be seen that Brazil recorded the highest mean stock index of 0.0556 (5.56%) with the highest standard deviation of 0.2612. This makes Brazil's market highly volatile, giving the reason for the widespread of variation. Turkey followed with a mean of 0.0241 and a standard deviation of 0.1078. India recorded a mean of 0.111 and a standard deviation of 0.0678. However, UK and US recorded the least values for standard deviation (0.0371 and 0.0375) respectively. The approximate value of standard deviation for UK and US makes their market index less volatile. Also, based on the means and standard deviation of UK and US, the stock market of US appears to be more attractive than that of the UK.

### 4.2 Spectral non-parametric seasonality test

The results of seasonality tests based on differenced data for all the countries are presented by type of market in Table 2 whiles the results based on the undifferenced data are presented by type of market in Table 3. As indicated earlier, the null distribution for a complete test against stochastic seasonality at all frequencies is the Cramér–von Mises distribution on 11 degrees of freedom with a critical value of 0.463. Values of the test statistic greater than 0.463 implies a significant test. It is obvious from Table 2, Table 3 that at both lags (m=4 and m=8), the same test decision is reached. From Table 2 we see that apart from Brazil which recorded stochastic seasonality at both lags, the seasonality of all the other markets is stationary. This demonstrates that one emerging market out of the nine [9], recorded stochastic seasonality while none of the industrial markets recorded stochastic seasonality. However, Fisher's exact test for equal proportions resulted in a p-value of 0.5 indicating no significant difference in proportion of stochastic seasonality between the two market types.

**Table 2:** Spectral Non-Parametric Seasonality Test by Type of Market (differenced data).

Emerging Market				Industrialized Market			
*Country*	Test Statistic (ω)		Test Status	*Country*	Test Statistic (ω)		Test Status
	*m* = 4	*m* = 8			*m* = 4	*m* = 8	
Brazil	1.2558	1.2645	Sig.	Austria	0.0532	0.0537	Not Sig.
India	0.0725	0.0732	Not Sig.	Canada	0.0303	0.0307	Not Sig.
Ireland	0.0421	0.0426	Not Sig.	Denmark	0.0330	0.0332	Not Sig.
Israel	0.0431	0.0434	Not Sig.	France	0.0374	0.0378	Not Sig.
Korea	0.0610	0.0614	Not Sig.	Germany	0.0386	0.0389	Not Sig.
Mexico	0.0579	0.0587	Not Sig.	Italy	0.0545	0.0538	Not Sig.
Portugal	0.0344	0.0347	Not Sig.	Japan	0.0404	0.0409	Not Sig.
S. Africa	0.0345	0.0340	Not Sig.	Netherlands	0.0358	0.0362	Not Sig.
Turkey	0.1855	0.1879	Not Sig.	Switzerland	0.0275	0.0277	Not Sig.
				UK	0.0265	0.0267	Not Sig.
				USA	0.0245	0.0248	Not Sig.

**Table 3:** Spectral non-parametric seasonality test by type of market.

Emerging Market				Industrialized Market			
*Country*	Test Statistic (*w*)		Test Status	*Country*	Test Statistic (*w*)		Test Status
	*m* = 4	*m* = 8			*m* = 4	*m* = 8	
Brazil	0.6712	0.6281	Sig.	Austria	0.0317	0.0315	Not Sig.
India	0.0473	0.0472	Not Sig.	Canada	0.0169	0.0170	Not Sig.
Ireland	0.0269	0.0263	Not Sig.	Denmark	0.0190	0.0184	Not Sig.
Israel	0.0260	0.0258	Not Sig.	France	0.0219	0.0219	Not Sig.
				Germany	0.0235	0.0231	Not Sig.
				Italy	0.0306	0.0302	Not Sig.

As indicated earlier, to ascertain Busetti and Harvey's [28] position that taking the first difference is likely to be a good strategy for testing the presence of stochastic seasonality in most economic time series, the study used the non-differenced data of ten markets (four emerging markets and six industrial markets) to carry out the seasonality tests. Table 3 displays the results of the tests which includes values of the test statistics at lags m=4 and m=8 together with the corresponding test status by market and type of market. It is clear from Table 2, Table 3 that, for the markets involved, the same test conclusions are observed even though the tests based on the non-differenced data recorded correspondingly lower estimates of test statistic values.

### 4.3 Test for January effect

The test for January effect was carried out for each of the twenty selected markets by means of the simple regression method with the response variable being the investment returns and the predictor variable which assumes the value 1 for January and zero otherwise. Table 4, Table 5 presents the results of the tests which include estimates of the slope parameter, the corresponding standard error, test statistics and p-values for the emerging markets and industrial markets respectively.

**Table 4:** Test for January effect (emerging markets).

Country	Coefficient	Standard error	*t*-test	p-value
Brazil	0.1915	0.0481	3.9810	0.0001
India	0.0085	0.0127	0.6670	0.5055
Ireland	0.0274	0.0098	2.8070	0.0053
Israel	0.0165	0.0095	1.7310	0.0843
Korea	0.0114	0.0118	0.9690	0.3330
Mexico	0.0067	0.0113	0.5980	0.5504
Portugal	0.0218	0.0089	2.4460	0.0149
South Africa	0.0172	0.0083	2.0890	0.0374
Turkey	0.0595	0.0200	2.9730	0.0032

**Table 5:** Test for January effect (industrialized markets).

Country	Coefficient	Standard error	*t*-test	p-value
Austria	0.0199	0.0106	1.8770	0.0612
Canada	0.0076	0.0076	0.9980	0.3190
Denmark	0.0282	0.0082	3.4290	0.0007
France	0.0118	0.0088	1.3460	0.1790
Germany	0.0134	0.0091	1.4680	0.1430
Italy	0.0301	0.0103	2.9210	0.0037
Japan	−0.0011	0.0092	−0.1190	0.9060
Netherlands	0.0139	0.0087	1.6040	0.1100
Switzerland	0.0090	0.0075	1.2050	0.2289
United Kingdom	0.0080	0.0070	1.1530	0.2500
United States	0.0041	0.0070	0.5860	0.5583

Irrespective of type of market, only the Japanese market recoded a negative (insignificant) January effect (Table 4). All the other markets recorded positive January effect even though not all are significant. Five of the emerging markets under investigation recorded positive significant January effect at 5% level of significance (Table 4): the markets together with their p-values are namely, Brazil (0.0001), Ireland (0.0053), Portugal (0.0149) South Africa (0.0374) and Turkey (0.0032).

It is also obvious from Table 5 that out of the eleven [11] industrial markets only two, Denmark (0.0007) and Italy (0.0037) recorded significant positive January effect. These implies that in all, seven [7] out of twenty [20] countries recorded a seasonal increase in stock prices throughout the month of January. The emerging market recorded more countries having the January effects against only 2 countries in Industrial countries. South Africa, Turkey, Portugal, Ireland and Brazil recorded an increase in the number of stockholders in the month of January alone for the emerging countries. Also, deductions from the Industrial countries indicated that Italy and Denmark had increased number of stockholders for the month of January. The Fisher's exact test for equal proportions, however, resulted in a p-value of 0.1749 indicating no significant difference in proportion of significant January effect between the two market types.

### 4.4 Test for market efficiency

This section describes the concept of efficiency applied to the stock market. As mentioned early on, the variance ratio test and the runs test were used to determine market efficiency. The variance ratio test assesses whether the returns follow the random walk theory while the runs test determines whether the returns are random. Significant test result for each of these tests implies an efficient market. Table 6, Table 7 display the test results estimate of variance ratio, test statistic and p-value for the variance ratio test and runs, test statistic value and p-value for the runs test by country for emerging and industrial markets respectively. It is clear from Table 6 that for both tests, all the emerging markets recorded p-values less than 0.05 indicating significant test results at 5% significance level. This means that all the emerging markets under investigation are efficient.

**Table 6:** Results of tests for market efficiency (emerging markets).

Country	Variance Ratio Test			Runs Test		
	Ratio	Test Stat.	*P*-val.	Runs	Test Stat.	*P*-val.
Brazil	4.8329	39.9988	0.0000	147	−4.1015	0.0000
India	4.1506	16.2232	0.0001	166	−2.1286	0.0333
Ireland	5.4149	19.9401	0.0000	153	−3.4785	0.0005
Israel	4.6841	19.2231	0.0000	165	−2.2325	0.0256
Korea	3.5552	14.1909	0.0002	140	−4.8283	0.0000
Mexico	4.1821	18.4422	0.0000	161	−2.6478	0.0081
Portugal	5.6297	32.0082	0.0000	142	−4.6207	0.0000
S. Africa	3.4889	9.4468	0.0021	151	−3.6861	0.0002
Turkey	3.8933	10.8584	0.0010	153	−3.4785	0.0005

**Table 7:** Results of tests for market efficiency (industrialized markets).

Country	Variance Ratio Test			Runs Test		
	Ratio	Test Stat.	*P*-val.	Runs	Test Stat.	*P*-val.
Austria	4.6772	8.5734	0.0034	149	−3.8938	0.0001
Canada	3.3688	8.8568	0.0029	162	−2.544	0.0110
Germany	3.8810	17.2779	0.0000	149	−3.8938	0.0001
Denmark	4.7892	10.4106	0.0013	171	−1.6094	0.1075
France	3.4186	19.0419	0.0000	152	−3.5823	0.0003
Italy	2.9490	13.2565	0.0003	151	−3.6861	0.0002
Japan	4.2355	20.9538	0.0000	140	−4.8283	0.0000
Netherlands	4.1976	12.6237	0.0004	150	−3.7900	0.0002
Switzerland	4.0046	12.0762	0.0005	167	−2.0248	0.0429
UK	1.3667	8.1686	0.0043	170	−1.7133	0.0867
USA	3.3425	8.6278	0.0033	168	−1.9210	0.0547

A slightly different story can be told about the industrialized markets based on the results in Table 7. While based on the variance ratio tests, all the industrial markets are efficient (p-values less than 5%) just like those of the emerging markets, the story is different under the runs test. Results from the runs test (Table 7) show that the markets of Denmark (0.1075), UK (0.0867) and USA (0.0547) are not efficient as they record p-values less than 5%. Thus, while all the nine [9] emerging markets are efficient under the period of investigation, seven out of the eleven [11] industrial markets are efficient. However, the Fisher's exact test for equal proportions resulted in a p-value of 0.1053 indicating no significant difference in proportion of market efficiency between the two market types.

## 5 Conclusions

This study sought to do comparative assessment of seasonality status, January effect status and efficiency status of emerging and industrial markets. The results of the spectral nonparametric tests showed that only Brazil (an emerging market) recorded significant test result indicating a stochastic seasonal return for the Brazilian market. These findings are in line with the position of Li et al. [26] that the stochastic seasonality trend of stock returns is economically significant in developed markets than emerging markets. However, the returns of all the other markets (irrespective of type) are not stochastically seasonal. Hence one needs to be careful when modelling the returns of the Brazilian market as opposed to those of the others. The results of the Fisher's test for equal proportion of markets with stochastic seasonal status was insignificant implying no difference between the two types of markets.

The seasonality tests based on the non-differenced data provided the same conclusions even though they resulted in lower estimates of test statistic values than those based on the differenced data. These go to buttress Busetti and Harvey's [28] assertion that taking the first difference is likely to be a good strategy for testing for the presence of stochastic seasonality because, on the borderline, the results could have been different.

With regards to January effect, five out of the nine [9] emerging markets, namely Brazil, Ireland, Portugal, South Africa and Turkey presented with the effect over the study period while only two of the industrial markets (Denmark and Italy) recorded the effect. The result is in conformity with Addinpujoartanto [12] that there is an existence of January effect in the stock returns of Indonesia. However, the January Effect was not found in small-company stock, except in large-company stock. Also, a study by Shen et al. [52] reported a January effect on the Taiwan Stock Exchange and concluded that the January effect is common in emerging markets as compared to industrial markets. The Fisher's exact test for equal proportions of markets with January effect, stated early on, however, didn't indicate a significant difference between the two types of market.

The variance ratio test and the runs test were used, to assess the market efficiency status of the two types of markets. Based on the results of the variance ratio tests, all the markets, irrespective of type, were shown to be efficient over the period of investigation. The run tests, however, produced a slight discrepancy. They recorded three inefficient markets (Denmark, UK and USA) among the industrial markets whiles all other markets (irrespective of type) are efficient. However, Fisher's exact test resulted in no difference between the two types of markets in terms of the proportion of efficient markets, supporting the conclusion based on the variance ratio tests.

Obviously, almost all the markets (19 out of 20) do not present stochastic seasonality over the study period, a majority (13 out of 20) had no January effect and almost all the markets are efficient. These support Kampman [53] assertion that one of the most common patterns of market efficiency anomalies is the seasonality effect, which comprises a variety of time-related impacts. The only market (Brazil) which presented stochastic seasonality also recorded the highest volatility (standard deviation) over the period of study, causing one to conclude that high volatility implies stochastic seasonality.

In view of the findings, the study concludes that there are no significant differences between emerging and industrialized markets in terms of seasonality status, January effect status, and market efficiency status. In this sense, the paper suggests a further study which considers more markets from both types of market and carries out a comparative analysis of the results from parametric and nonparametric tests for the presence of stochastic seasonality, January effect and market efficiency.

## Data availability statement

Data will be available upon request from the corresponding author.

## Funding

There was no funding for this manuscript.

## CRediT authorship contribution statement

**Nancy Eduah:** Writing – original draft, Conceptualization. **Godwin Debrah:** Writing – review & editing, Supervision. **Emmanuel Kojo Aidoo:** Software, Formal analysis, Data curation. **Felix O. Mettle:** Supervision, Methodology, Conceptualization.

## Declaration of competing interest

The authors declare that they have no known competing financial interests or personal relationships that could have appeared to influence the work reported in this paper.

## Figures and Tables

**Figure:**